# Buying Difficulties of the Present Day

**Published:** 1921-08-06

**Authors:** 


### THE HOSPITAL COMMISSARIAT.

### Buying Difficulties of the Present Day.

One looks back with regret to the days before the war, when, as a rule, a hospital made use of a number of tradesmen who were rarely changed, and from them obtained supplies on a six or twelve months' contract at prices which varied but little from year to year. It was a simple thing when contract time came round to tabulate the various quotations, showing exactly what the requirements of the hospital would cost during a given period according to the quotations of those who had submitted tenders. The carrying out of the contracts was also uneventful. As is usual, the conditions were often remarkably well observed during the first part and the last part of the term. There were a certain number of grumbles during the period, but, after a sharp letter or two had been written to him, the contractor was again chosen when the matter came up for the consideration of the Board. The war brought an end to this happy state of things, and after it had been in progress for a very few months contracts of any kind were difficult to make. The only possible ones were so hedged round by conditions as to variation in prices, according to the decisions of the Food Controller or other Government powers, that in effect they were just as troublesome as dealing in the open market. There was, however, this benefit, that most of the hospitals stuck to their old tradesmen, and tradesmen stuck to their old customers, securing thereby some kind of supply.

### When Contracts are Undesirable.

After the close of the war it was hoped that things would revert to the former easy ways, but it was by no means early after the Armistice that anything in the shape of a satisfactory contract was obtainable. Within the last year or two there has been in respect of many articles of food a falling market, and we understand that some of the larger hospitals take the view that it is not a business-like proposition to make contracts in such a period. On the other hand, there are quite a number of large and important institutions which continue to contract. We believe that at the Middlesex Hospital, for instance, a monthly contract for meat, fish, milk, provisions, and groceries is made, while elsewhere contracts from three to six months are the general rule. Periods beyond six months are not, as in pre-war times, ventured upon. The thing that a hospital aims at chiefly is to obtain supplies of a uniformly good character at a fair market rate, and as to whether this is better obtained by contracting or by buying from day to day in the market there is considerable difference of opinion. From the administrative point of view there is little doubt that a contract is most desirable, as the large amount of vigilance required in daily buying and a large part of the work of checking accounts are with it no longer necessary.

### Milk and Eggs.

There is one article in which buying is remarkably simple, and that is milk. The only question of competition which enters into this supply, taking it that quality is equal in all cases, is how much the wholesaler will charge below the retail price of the neighbourhood. This varies from 6d. to 10d. a gallon. Naturally, in the matter of milk supplied to a public institution, quality must be assured, but in London at present, if not elsewhere, there is sufficient competition to protect the institution in the matter of prices. Another article in which it is common experience that a contract, at least at present, is not desirable, is eggs. Eggs cost much about the same whether bought by contract or from day to day, and the best plan is for a hospital to arrange to have first call upon the output of a certain farm, stipulating for eggs of a certain weight or size. In a contract, origin and degrees of freshness are difficult to govern. The question of price is easily checked by periodical inquiries in different quarters.

With the exception of potatoes it has never been possible to make a satisfactory contract for vegetables. Happy is the hospital that has its country branch or convalescent home which can help in this matter. Others are forced to buy at what are practically full retail prices from the local shops, and, short of employing someone to visit the markets from day to day, there seems no help for what appears to be an indolent and unimaginative solution of the difficulty. Fish can be bought by contract possibly better than by day-to-day purchasing. First, it is necessary to get the contractor to understand the hospital system of diets, under which the supplies are delivered dressed, cut and cleaned, ready for table, in pieces of a certain weight. Large numbers of contracts for fish are made direct with Grimsby, but the hospital has to stand the risk of railway breakdown or other bars to delivery.

Tea is an article which is best bought direct from a firm according to requirements. Both prices and quality are easily checked, and supplies can be obtained and stored for forward use for some months. In the purchase of some other articles, such as provisions, the facilities offered by the large stores should be carefully investigated. We have before us an instance of an institution breaking its contract because of unsatisfactory quality in supply in the matter of ham; 2s. 1d. per lb. was paid under the contract, and a better article was obtained from one of the large stores at 1s. 10½d. per lb.

### Co-operative Buying and its Difficulties.

The report of Lord Cave's Committee hints at a possibility of co-operation in buying. The only experiment that has been made in London was discontinued upon its being found that there was little or no advantage. It is true that the experiment was conducted on lines which were not very promising. The whole arrangement may be summed up by saying that there was a possibility of small institutions having the advantage of the same terms as one of the larger of our Metropolitan hospitals, who retained the right to make all decisions in respect of the contract. This in many respects was plainly not co-operative buying. In any case, co-operative buying in its fullest sense would need something possibly substantial in the way of extra administration, and might possibly also mean a central storehouse for non-perishable supplies. These are subjects which require careful consideration from those that they affect.

